# Comparative efficacy of Neuraxial and general anesthesia for hip fracture surgery: a meta-analysis of randomized clinical trials

**DOI:** 10.1186/s12871-020-01074-y

**Published:** 2020-06-30

**Authors:** Xinxun Zheng, Yuming Tan, Yuan Gao, Zhiheng Liu

**Affiliations:** Department of Anesthesiology, Shenzhen Second People’s Hospital, The First Affiliated Hospital of Shenzhen University, Shenzhen, 518000 China

**Keywords:** Neuraxial anesthesia, General anesthesia, Hip fracture

## Abstract

**Background:**

The choice of anesthesia technique remains debatable in patients undergoing surgical repair of hip fracture. This meta-analysis was performed to compare the effect of neuraxial (epidural/spinal) versus general anesthesia on perioperative outcomes in patients undergoing hip fracture surgery.

**Methods:**

Medline, Cochrane Library, Science-Direct, and EMBASE databases were searched to identify eligible studies focused on the comparison between neuraxial and general anesthesia in hip fracture patients between January 2000 and May 2019. Perioperative outcomes were extracted for systemic analysis. Sensitivity analyses were conducted using a Bonferroni correction and the leave-one-out method. The evidence quality for each outcome was evaluated by the Grading of Recommendations Assessment, Development and Evaluation (GRADE) system.

**Results:**

Nine randomized controlled trials (RCTs) including 1084 patients fulfilled our selection criteria. The outcomes for the meta-analysis showed that there were no significant differences in the 30-day mortality (OR = 1.34, 95% CI 0.56, 3.21; *P* = 0.51), length of stay (MD = − 0.65, 95% CI -0.32, 0.02; *P* = 0.06), and the prevalence of delirium (OR = 1.05, 95% CI 0.27, 4.00; *P* = 0.95), acute myocardial infarction (OR = 0.88, 95% CI 0.17, 4.65; *P* = 0.88), deep venous thrombosis (OR = 0.48, 95% CI 0.09, 2.72; *P* = 0.41), and pneumonia (OR = 1.04, 95% CI 0.23, 4.61; *P* = 0.96) for neuraxial anesthesia compared to general anesthesia, and there was a significant difference in blood loss between the two groups (MD = − 137.8, 95% CI -241.49, − 34.12; *p* = 0.009). However, after applying the Bonferroni correction for multiple testing, all the adjusted *p*-values were above the significant threshold of 0.05. The evidence quality for each outcome evaluated by the GRADE system was low.

**Conclusions:**

In summary, our present study demonstrated that there might be a difference in blood loss between patients receiving neuraxial and general anaesthesia, however, this analysis was not robust to adjustment for multiple testing and therefore at high risk for a type I error. Due to small sample size and enormous inconsistency in the choice of outcome measures, more high-quality studies with large sample size are needed to clarify this issue.

## Background

Hip fracture is one of the most common injuries that occurs in about 1.6 million people around the world each year; the number is estimated to reach more than six million by 2050 [1]. Moreover, there are a range of comorbidities in elderly patients with hip fracture, which are associated with an increased risk of morbidity and mortality [2]. Most hip fractures should be treated surgically that requires some type of anesthesia [3].

Thus far, the ideal choice between neuraxial and general anesthesia has not been identified. Several studies demonstrated that compared with general anesthesia, neuraxial anesthesia has some advantages such as airway management avoidance, no intubation requirement, and prolonged postoperative analgesia [4]. Furthermore, neuraxial anesthesia could decrease blood loss, potentially reduce risk of postoperative nausea and vomitting (PONV), as well as deep venous thrombosis [4–6]. Conversely, general anesthesia is reported to provide a more stable hemodynamic state, faster induction, and avoid some complications such as pneumonia, epidural haematoma and infection [7, 8]. However, the effect of the two anesthesia techniques on patients with hip fracture is controversial regarding postoperative outcomes. A recent systematic review including 15 studies revealed that neuraxial anesthesia was only associated with a shorter length of hospital stay in patients undergoing hip fracture surgery. This review emphasized that sensitivity analyses showed marginal statistical significance for length of stay favoring spinal anaesthesia, and the definitions of reported outcomes varied widely or were unclear, making evaluation in a standardized manner very difficult [9]. Another systematic review reported a reduced in-hospital mortality in the neuraxial anaesthesia group, but no definitive conclusion can be drawn for longer-term mortality [10]. Both of them have recommended that further high-quality studies be performed.

To date, several most recent randomized controlled trials (RCTs) have been published, which assessed the effect of the two anesthesia techniques for hip fracture surgery. Through including these RCTs, our study aimed to systematically evaluate perioperative outcomes of patients with hip fracture surgery, and provide more reliable evidence to identify the optimal technique.

## Methods

This meta-analysis was conducted in accordance with Preferred Reporting Items for Systematic Reviews and Meta-analyses (PRISMA) guidelines. It was registered in the international prospective register of systematic reviews (Prospero: CRD42020143172).

### Search strategy

Medline, Cochrane Library, Science-Direct, and EMBASE databases were searched by two independent reviewers between January 2000 and May 2019. We selected studies of neuraxial anesthesia compared with general anesthesia in patients undergoing hip fracture surgery. Following iterms were searched for both alone and in various combinations, “hip fracture” or “femur fracture” or “intertrochanteric” or “femoral neck” AND “regional anesthesia” or “spinal anesthesia” or “neuraxial anesthesia” or “epidural anesthesia”. The “related articles” function in Medline was performed to expand the search. Reference lists were also hand-searched for relevant studies. No language restriction was placed on our search.

### Inclusion and exclusion criteria

Two independent reviewers screened article titles and abstracts based on the following inclusion criteria: (1) randomized controlled trials (RCTs) with no language restriction; (2) studies comparing general anesthesia with neuraxial anesthesia (epidural or spinal) in patients undergoing hip fracture surgery; (3) studies provided numerical data. The following exclusion criteria was used: (1) studies that did not meet the inclusion criteria; (2) unpublished data or repeated data; (3) abstracts, case reports, comments, conference papers, or animal studies, meta-analysis and systematic reviews.

### Data extraction

Two independent reviewers designed a structured table and collected all the relevant data into a database. The following information was extracted from each study that met the inclusion criteria: first author’s name, publication year, country, sample size, age, American Society of Anesthesiologists (ASA) physical status, anesthesia technique, surgery type, study outcome measures. We also attempted to contact the corresponding authors to verify the accuracy of the data and to obtain further analytical data. We performed a meta-analysis for blood loss, 30-day mortality, length of hospital stay, and the prevalence of delirium, acute myocardial infarction, deep venous thrombosis, and pneumonia.

### Methodological quality assessment

The methodological quality of each RCT was assessed using the Cochrane Handbook for Systematic Reviews of Interventions 5.1 by two reviewers, which contained the following items: random sequence generation, allocation concealment, blinding, incomplete outcome data, selective reporting, and other sources of bias. It was judged by answering a question, with “yes” indicating low risk of bias, “no” indicating high risk of bias, and “unclear” indicating unclear or unknown risk of bias [11]. The corresponding author was also consulted when any disagreement exists, and a consensus was reached by discussion.

### Statistical analysis

The statistical analysis of the pooled data were performed using Review Manager software (version 5.1, The Cochrane Collaboration, Oxford, England). For continuous variables, standardized mean difference (SMD) or weighted mean (WMD) difference was calculated with the 95% confidence intervals (CIs) as a summary statistic. For dichotomous variables, relative risk (RR) and 95% CIs were used. The combined effect was considered significant at a 2-sided *P* < 0.05. The *p*-value with the Cochrane Q-test was texted, and the I^2^ statistic was used to judge inconsistency of treatment effects across studies. A random effect model was used if high heterogeneity was detected (*p* < 0.10, I^2^ > 50%); otherwise, a fixed effect model was used if low heterogeneity existed (*p* > 0.10, I^2^ < 50%). Sensitivity analyses included a Bonferroni correction to adjust for multiple testing as well as the leave-one-out method. Publication bias was evaluated by funnel plot, if our meta-analysis included more than 10 studies [12].

### Evidence synthesis

The evidence grade for the main outcomes are assessed using the guidelines of the (GRADE) system working group including the following items: risk of bias, inconsistency, indirectness, imprecision and publication bias. The recommendation level of evidence is classified into the following categories: (1) high, which means that further research is unlikely to change confidence in the effect estimate; (2) moderate, which means that further research is likely to significantly change confidence in the effect estimate but may change the estimate; (3) low, which means that further research is likely to significantly change confidence in the effect estimate and to change the estimate; and (4) very low, which means that any effect estimate is uncertain. The evidence quality is graded using the GRADEpro Version 3.6 software. The evidence quality was graded using the GRADEpro Version 3.6 software. The strengths of the recommendations were based on the quality of the evidence.

## Results

### Study identification and selection

**Fig. 1:**
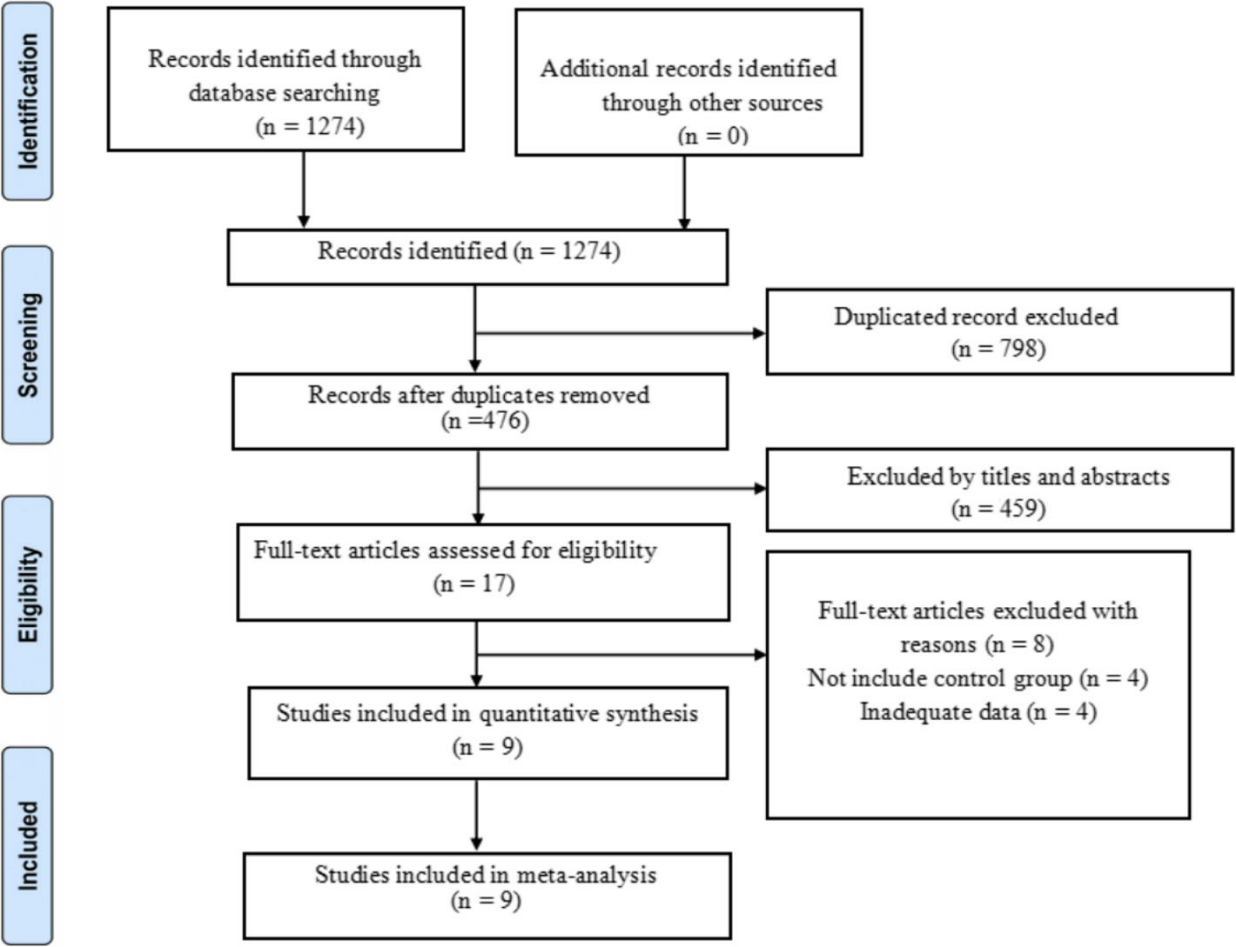
The flow diagram of study selection

A total of 1274 relevant studies were identified according to the search strategy. However, 798 publications were excluded after checking for duplicates. Among the 476 remaining articles, 359 articles were excluded after reviewing the titles and abstracts. Then we assessed 17 studies with full texts for eligibility. Eight studies were excluded because four of them included no control groups, and others provided inadequate data. Finally, nine RCTs with a total of 1084 patients between 2003 and 2018 met our inclusion criteria, and were included in the meta-analysis [13–21]. The flow diagram of study selection is shown in Fig. 1.

### Study characteristics

**Fig. 2:**
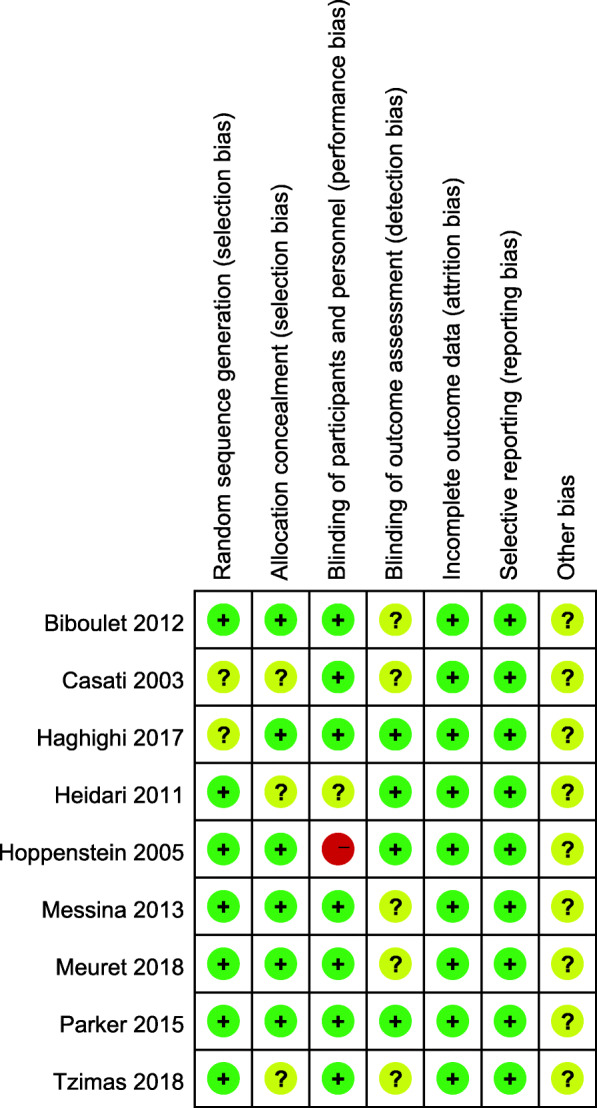
Summary of risk bias assessment. “+” = low risk of bias; “?” = unclear risk of bias; and “-” = high risk of bias

All the included studies were written in English, which examined perioperative outcomes between hip fracture patients who receive neuraxial or general anesthesia undergoing surgical repair. There were a total of 1084 patients, whose ages were older than 49 years-old. Seven studies looked at outcomes relating to spinal anesthesia compared with general anesthesia [13, 14, 16–19, 21], one study examed outcomes for hypobaric unilateral spinal anesthesia and general anesthesia [20], and the other study compared general versus neuraxial anesthesia that encompassed spinal and epidural anesthesia [15]. In the terms of surgery type, two studies performed arthroplasty, hip screw and intramedullary nail [18, 20]; two studies included hemiarthroplasty only [13, 14], and one study performed hemiarthroplasty and Intramedullary nail [16]. Only one study was at a high risk of performance bias [14], and the other studies were all at low risk or unclear (Fig. 2). The characteristics of the included studies is shown in Table 1.

**Table 1:** The descriptive characteristics of included studies

Study	Country	Sample size (male/female)	Age in years	ASA status	anesthesia	surgery type	Study outcome measures
Casati et al. (2003) [13]	Italy	30 (7/23)	84	II-III	GA vs Spinal	Hemiarthroplasty	Delirium; MMSE
Hoppensteinet al. (2005) [14]	Israel	60	> 65	I-III	GA vs Spinal	Hemiarthroplasty	Hemodynamic change; Delirium
Heidariet al. (2011) [15]	Iran	387 (257/130)	> 60	I-III	GA vs NA	–	Length of stay; 30-day mortality; Acute myocardial infarction; Pneumonia; Blood loss
Biboulet et al. (2012) [16]	France	45 (14/31)	> 75	III-IV	GA vs Spinal	Hemiarthroplasty Intramedullary nail	30-day mortality; Acute myocardial infarction
Messina et al. (2013) [17]	Italy	20 (7/13)	> 75	III	GA vs Spinal	–	Blood loss; Hemodynamic change
Parker et al. (2015) [18]	UK	322 (87/235)	> 49	I-III	GA vs Spinal	Arthroplasty Sliding hip screw Intramedullary nail	Delirium; 30-day mortality Acute myocardial Infarction; Pneumonia; Length of stay; DVT
Haghighi et al. (2017) [19]	Iran	100 (80/20)	> 60	I-III	GA vs Spinal	–	Blood loss; PONV
Meuret et al. (2018) [20]	France	40 (8/32)	> 75	I-III	GA vs HUSA	Arthroplasty Dynamic hip screw Intramedullary nail	PONV; DVT
Tzimas et al. (2018) [21]	Greece	70 (33/37)	76	I-III	GA vs Spinal	–	Delirium; MMSE

*MMSE* mini mental state examination, *PONV* post operative nausea and vomitting, *GA* general anesthesia, *NA* neuraxial anesthesia, *ASA* American Society of Anesthesiologists, *HUSA* hypobaric unilateral spinal anesthesia, *DVT* deep venous thrombosis

### Outcomes for meta-analysis

**Fig. 3:**
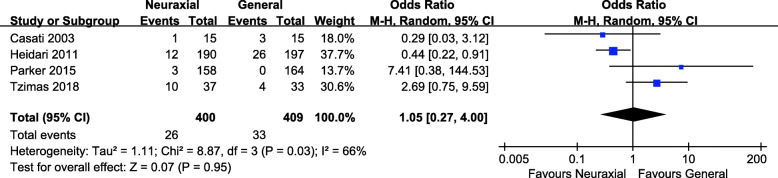
Forest plot of delirium rate for neuraxial anesthesia versus general anesthesia

Delirium rate was reported in four studies with 400 patients in the neuraxial anesthesia group and 409 patients in the general anesthesia group [13, 15, 18, 21]. The *P* value with the Cochran’s Q test was 0.03, and the I^2^ statistic was 66%, which indicated high heterogeneity among these studies. Thus a random effect model was used to analyze the results. The pooled data showed no significant difference in delirium rate between the two groups (OR = 1.05, 95% CI 0.27, 4.00; *P* = 0.95, Fig. 3).

**Fig. 4:**
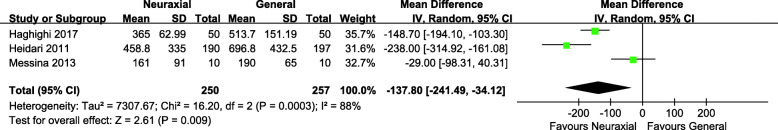
Forest plot of blood loss for neuraxial anesthesia versus general anesthesia

Three studies examed blood loss during hip fracture surgery with 250 patients in the neuraxial anesthesia group and 257 patients in the general anesthesia group [15, 17, 19]. The *P* value with the Cochran’s Q test was 0.0003, and the I^2^ statistic was 88%, which indicated high heterogeneity among these studies. Thus a random effect model was used to analyze the results. The pooled data showed a significant difference between the two groups (MD = − 137.8, 95% CI -241.49, − 34.12; *p* = 0.009, Fig. 4).

**Fig. 5:**
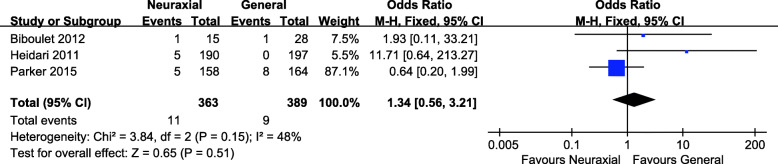
Forest plot of 30-day mortality for neuraxial anesthesia versus general anesthesia

Three studies were included in the meta-analysis for 30-day mortality, involving 363 patients in the neuraxial anesthesia group and 389 patients in the general anesthesia group [15, 16, 18]. The *P* value with the Cochran’s Q test was 0.21, and the I^2^ statistic was 48%, which indicated low heterogeneity among these studies. Thus a fixed effect model was used to analyze the results. The pooled data revealed that there was no significant difference in 30-day mortality between the two groups (OR = 1.34, 95% CI 0.56, 3.21; *P* = 0.51, Fig. 5).

**Fig. 6:**
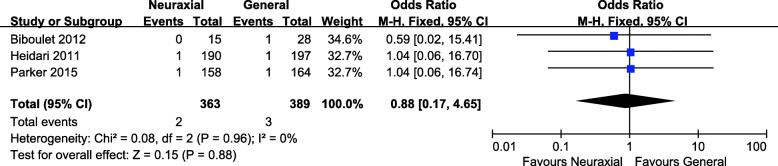
Forest plot of acute myocardial infarction rate for neuraxial anesthesia versus general anesthesia

Acute myocardial infarction rate was reported in three studies with 363 patients in the neuraxial anesthesia group and 376 patients in the general anesthesia group [15, 16, 18]. The *P* value with the Cochran’s Q test was 0.96, and the I^2^ statistic was 0%, which indicated low heterogeneity among these studies. Thus a fixed effect model was used to analyze the results. The pooled data showed no significant difference in the acute myocardial infarction rate between the two groups (OR = 0.88, 95% CI 0.17, 4.65; *P* = 0.88, Fig. 6).

**Fig. 7:**
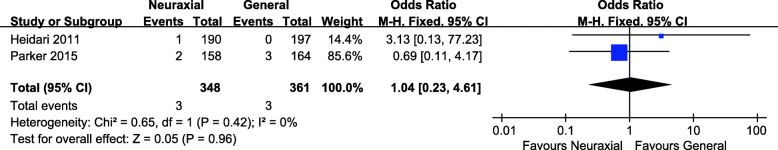
Forest plot of pneumonia rate for neuraxial anesthesia versus general anesthesia

Two studies provided the outcome of pneumonia rate, which involved 363 patients in the neuraxial anesthesia group and 389 patients in the general anesthesia group [15, 18]. The *P* value with the Cochran’s Q test was 0.42, and the I^2^ statistic was 0%, which indicated low heterogeneity among these studies. Thus a fixed effect model was used to analyze the results. The pooled data showed no significant difference in pneumonia rate between the two groups (OR = 1.04, 95% CI 0.23, 4.61; *P* = 0.96, Fig. 7).

**Fig. 8:**
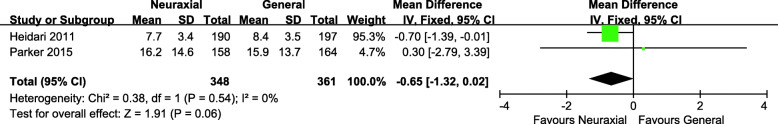
Forest plot of length of stay for neuraxial anesthesia versus general anesthesia

Two studies reported length of stay in a way that could be comparable by meta-analysis, including 348 patients in the neuraxial anesthesia group and 361 patients in the general anesthesia group [15, 18]. The *P* value with the Cochran’s Q test was 0.54, and the I^2^ statistic was 0%, which indicated low heterogeneity among these studies. Thus a fixed effect model was used to analyze the results. The pooled data revealed that no significant difference was detected in the length of stay between the two groups (MD = − 0.65, 95% CI -0.32, 0.02; *P* = 0.06, Fig. 8).

**Fig. 9:**
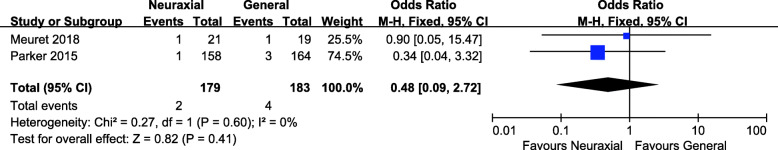
Forest plot of deep venous thrombosis rate for neuraxial anesthesia versus general anesthesia

Two studies were included in the meta-analysis for deep venous thrombosis rate, involving 179 patients in the neuraxial anesthesia group and 183 patients in the general anesthesia group [18, 20]. The *P* value with the Cochran’s Q test was 0.60, and the I^2^ statistic was 0%, which indicated low heterogeneity among these studies. Thus a fixed effect model was used to analyze the results. The pooled data revealed that there was no significant difference in deep venous thrombosis rate between the two groups (OR = 0.48, 95% CI 0.09, 2.72; *P* = 0.41, Fig. 9).

### Sensitivity analysis

**Fig. 10:**
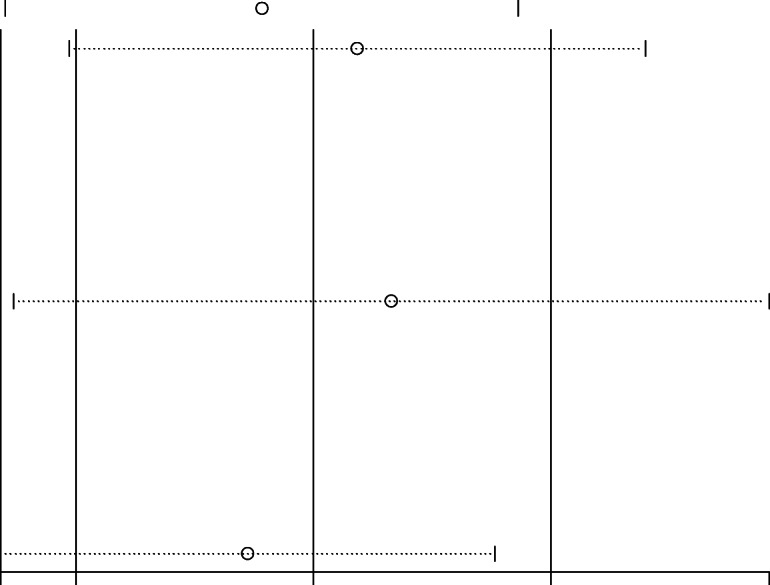
Sensitivity analysis of blood loss for neuraxial anesthesia versus general anesthesia

Sensitivity analyses were performed by the leave-one-out approach in the comparison of blood loss. No difference was detected in the direction of the outcome with each study removed in turn, which showed that this result had good reliability (Fig. 10).

After adjustment for multiple testing using the Bonferroni correction, adjusted *p*-values were 0.054 for the comparison of blood loss, 0.36 for the comparison of length of stay and 1.0 for the other outcomes. All of them were above the significant threshold of 0.05, thus there was no significant difference in each comparison.

### Quality of the evidence and recommendation strengths

A total of seven outcomes in this meta-analysis were evaluated using the GRADE system (Table 2). The evidence quality for each outcome was low. Therefore, we demonstrate that the overall evidence quality is low, which means that further research is likely to significantly change confidence in the effect estimate and may change the estimate.

**Table 2:** The GRADE evidence quality for main outcome

Quality assessment							No of patients		Effect		Quality	Importance
No of studies	Design	Risk of bias	Inconsistency	Indirectness	Imprecision	Other considerations	Experim-ental	Control	Relative (95% CI)	Absolute		
**Delirium rate**												
4	randomised trials	serious	no serious inconsistency	serious	no serious imprecision	none	26/400 (6.5%)	33/409 (8.1%)	OR 1.05 (0.27 to 4)	4 more per 1000 (from 58 fewer to 179 more)	⊕⊕**◯⃝**LOW	IMPORTANT
								12.7%		6 more per 1000 (from 89 fewer to 241 more)		
**Acute myocardial infarction rate**												
3	randomised trials	no serious risk of bias	serious	serious	no serious imprecision	none	2/363 (0.55%)	3/376 (0.8%)	OR 0.73 (0.14 to 3.74)	2 fewer per 1000 (from 7 fewer to 21 more)	⊕⊕**◯⃝**LOW	IMPORTANT
								0.6%		2 fewer per 1000 (from 5 fewer to 16 more)		
**30-day Mortality**												
3	randomised trials	serious	no serious inconsistency	no serious indirectness	serious	none	11/331 (3.3%)	16/343 (4.7%)	OR 0.71 (0.33 to 1.53)	13 fewer per 1000 (from 31 fewer to 23 more)	⊕⊕**◯⃝** LOW	IMPORTANT
								4.9%		14 fewer per 1000 (from 32 fewer to 24 more)		
**Blood loss (Better indicated by lower values)**												
3	randomised trials	serious	serious	no serious indirectness	no serious imprecision	none	250	257	–	MD −137.80 lower (−241.49 lower to −34.12 higher)	⊕⊕**◯⃝** LOW	IMPORTANT
**Pneumonia rate**												
2	randomised trials	serious	serious	no serious indirectness	no serious imprecision	reporting bias	3/348 (0.9%)	3/361 (0.8%)	OR 1.04 (0.23 to 4.61)	0 more per 1000 (from 6 fewer to 29 more)	⊕**◯⃝⃝** VERY LOW	NOT IMPORTANT
								0.9%		0 more per 1000 (from 7 fewer to 31 more)		
**Length of stay**												
2	randomised trials	serious	no serious inconsistency	serious	no serious imprecision	reporting bias	348	361	–	MD −0.65 lower (−1.32 lower to 0.02 higher)	⊕**◯⃝⃝** VERY LOW	NOT IMPORTANT
**Deep venous thrombosis rate**												
2	randomised trials	serious	no serious inconsistency	serious	no serious imprecision	None	2/179 (1.1%)	4/183 (2.2%)	OR 0.48 (0.09 to 2.72)	0 more per 1000 (from 2 fewer to 19 more)	⊕**◯⃝⃝** VERY LOW	NOT IMPORTANT
								2.3%		0 more per 1000 (from 6 fewer to 32 more)		

## Discussion

In our study, a total of nine RCTs with 1084 patients were included to make an updated meta-analysis. However, no significant difference was detected in the 30-day mortality, length of stay, and the prevalence of delirium, acute myocardial infarction, and pneumonia in patients undergoing hip fracture surgery where either neuraxial or general anesthesia was used. We first focused on the comparison of blood loss between the two anesthesia techniques. The leave-one-out method showed that the result had good reliability. However, after applying the Bonferroni correction, the adjusted *p*-value for this comparison was above the significance threshold (*p* = 0.054), which revealed there was no significant difference. The sample size was also small, and the overall evidence was low, indicating that further research is likely to significantly change confidence in the effect estimate and may change the estimate. Based on the current available evidence, more high-quality RCTs are required for further investigation.

According to methodological quality assessment, eight out of nine RCTs in our study were assessed as high-quality. Moreover, our study included several RCTs, in which the results were published after the most recent systematic review of this topic, making our results more dependable. Of note, all of the RCTs showed low risk of attrition bias and reporting bias that may contribute to reducing systematic bias. Another strength of our study is low heterogeneity, detected in five out of six outcome measures assessed using I^2^ statistic, demonstrating consistent outcomes across the comparisons. In addition, the adjustment was made by the Bonferroni correction to decrease the risk of type I error caused by multiple statistical tests in our study. Also, some data of previous reviews dated back to the 1980s [9, 10], in which the type of anaesthetic techniques may not reflect current clinical practice, and it may restrain us from finding clinically relevant differences between the two techniques [9, 22], while our study included most recent RCTs.

According to pharmacology, neuraxial anesthesia could lead to lower heart rate, and blood pressure than general anesthesia by blocking alpha and beta adrenergic receptors. Consequently, controlled blood pressure resulted in intraoperative less blood loss in neuraxial anesthesia patients [16, 23]. Current practice revealed that the number of patients who needed blood transfusion was larger in general anesthesia group, which means patients receiving spinal anesthesia had less blood loss than those receiving general anesthesia [19, 24, 25]. In consistency with this result, a systematic review by Richman et al. Including 66 articles demonstrated that the use of neuraxial anesthesia resulted in a significant decrease in estimated blood loss [26]. However, a meta-analysis by Hu er al. including 21 RCTs stated that there was insufficient evidence to support the use of neuraxial anesthesia in decreasing intraoperative blood loss [27]. In our study, only three RCTs involving 507 patients have been summarised. Two of them showed the neuraxial anesthesia was assosiated with statistically significant decrease in blood loss, the other showed no significant difference between the two anesthesia techniques. However, the results from our meta-analysis indicating decreased blood loss with neuraxial anesthesia are limited by a high degree of heterogeneity (88%) and low-quality evidence for this outcome. Also, we did not investigate whether this resulted in a clinically meaningful difference in perioperative blood transfusions.

Delirium is a very common postoperative complication, which leads to lasting cognitive and functional decline, and increasing length of stay [18, 28]. There are many precipitating factors in developing delirium, including infection, myocardial and cerebral ischaemia, urinary retention, pain, constipation as well as electrolyte abnormalities [29]. Furthermore, several studies have investigated the incidence of delirium in elderly patients, who were admitted to be hospitalized for a variety of reasons, and the prevalence amongst medical wards was estimated to range from 29 to 64% [29–31]. Additionally, the development of delirium is thought to be multifactorial process. Certain patient characteristics are also easy to cause delirium, including pre-existing cognitive impairment, sleep deprivation, medical immobilities, visual impairment, hearing impairment and poly pharmacy [32, 33]. Our study detected no significant difference in delirium rate between general and neuraxial anesthesia. It is noteworthy that none of the included studies represented relative characteristics and potential risk factors that causing delirium in hip fracture patients perioperatively. Thus the result may be unconvincing.

Our study detect comparable outcomes in the incidence of 30-day mortality between the two groups. In line with this result, a retrospective study reported that the anesthesia technique has little effect on postoperative mortality, and the type of anesthesia given by the anesthesiologist should be selected based on the individual physical condition [34]. The study of Lienhart et al. including 425 patients indicated that their coexisting disease has great influence on 30-day mortality in old patients such as diabetes, cardiovascular disease, etc [35]. Delay of surgery for more than 24 h was a main factor affecting postoperative mortality in geriatric hip fracture patients [36]. The retrospective cohort study of Pincus et al. Investigated 42,230 patients undergoing hip fracture surgery, and demonstrate that a preoperative waiting time of more than 24 h was associated with a greater risk of 30-day mortality and other complications [37].

In our study, the incidence of myocardial infarction and pneumonia were similar in both groups. Zuo et al. detected the same result, and suggested that the neuraxial anesthesia might be a better choice in hip fracture surgery [38]. However, Urwin et al. proposed that the incidence of myocardial infarction and pneumonia was lower in patients receiving neuraxial anesthesia, and a significant lower incidence of intraoperative hypotension was detected in patients receiving general anesthesia [39]. It should be noted that Urwin et al. evaluated 2161 patients retrospectively. Moreover, all of the included studies were performed more than 20 years ago, which are now somewhat dated, since many drugs used for anesthesia techniques and health care systems have been improved a lot. Thus their findings could not provide worthy references to some extent.

There was no significant difference regarding the length of stay between the two anesthesia techniques. Sutcliffe et al. surveyed 1333 volunteers of hip surgery, and found no difference in factors of hospitalization in both groups [40]. Neverthless, Neuman et al. conducted a matched retrospective cohort study involving 56,729 patients, and found a modestly shorter length of stay in the neuraxial anesthesia group. The authors also proposed that the fracture type and performed surgery procedure were important factors; minimally invasive approaches and optimal quality of fracture reduction may decrease the length of stay [41]. In addition, Grant et al. declared that the pain severity was lower in patients receiving general anesthesia, resuting in shorter length of stay [42]. A notable point is that waiting time prior to surgery extended the length of stay [43]. In our meta-analysis, one study reported the overall length of stay [18] while the other documented the length of stay before and after the surgery [15]. It is difficult to draw a definite conclusion due to the existence of aforementioned multiple factors. Also, the small sample size in our study should be taken into consideration.

Perioperative deep venous thrombosis is common in hip fracture patients. Several studies concluded that neuraxial anaesthesia was associated with fewer incidents of deep venous thrombosis when compared to general anaesthesia [39, 44, 45]. It was thought that in neuraxial anaesthesia sympathetic block could lead to vasodilatation of the lower limbs, and then the increased blood flow to the lower limbs was likely to reduce the coagulability and viscosity of blood [46]. A Cochrane review published in 2016 by pooling the results from 31 RCTs showed a reduced risk of deep venous thrombosis in the neuraxial group without potent thromboprophylaxis. Nevertheless, the level of evidence was very low for this outcome [22]. Another Cochrane review concluded that there was a marginal advantage for neuraxial anaesthesia regarding the incidence of deep venous thrombosis [47]. Our study included only two RCTs, and detected no significant difference in the incidence of deep venous thrombosis between the two groups.

Objectively speaking, several limitations of our study should be mentioned. A major limitation is that the sample size was relatively small, and the sample size varied widely among the included studies. Another notable limitation is that most of the included studies did not describe whether additional sedation was used in hip fracture patients receiving neuraxial anesthesia, for instance, the use of propofol sedation could influence the prevalence of postoperative delirium [48]. Also, no information is available in the terms of the dosage of the anesthesia used. In addition, the inconsistent definition of length of stay and delirium may account for the wide prevalence range for these outcomes. There are numerous confounding factors such as the diversity of patient groups, health care systems, surgical and anesthetic techniques that may affect the perioperative outcomes, leading to potential biases. This issue would be possibly considered as a weakness. Last but not least, the outcome measures were not identical in each trial, thus we did not have sufficient data to perform other meta-analyses, which potentially affects the current findings of our study. Therefore, more high-quality RCTs with large sample size are required for a firm conclusion.

## Conclusion

In summary, our present study demonstrated that there might be a difference in blood loss between patients receiving neuraxial and general anaesthesia, however, this analysis was not robust to adjustment for multiple testing and therefore at high risk for a type I error. We suggest that the choice of anaesthesia (neuraxial or general) should be made by the anaesthesiologist based on the individual patient’s requirements, comorbidities, potential postoperative complications, consultation of geriatrician and orthopaedic surgeon, and the clinical experience of the anaesthesiologist. Due to small sample size and enormous inconsistency in the choice of outcome measures, more high-quality studies with large sample size are needed to to clarify this issue.

## Data Availability

All data generated or analyzed during this study are included in this published article.

## Abbreviations

ASA: American Society of Anesthesiologists
CI: Confidence interval
GRADE: The grading of recommendations, assessment, development, and evaluation methodology
GA: General anesthesia
HUSA: Hypobaric unilateral spinal anesthesia
MMSE: Mini mental state examination
NA: Neuraxial anesthesia
PONV: Postoperative nausea and vomiting
PRISMA: Preferred reporting items for systematic reviews and meta-analyses
RCT: Randomized controlled trial
RR: Risk ratio
SMD: Standardized mean difference
WMD: Weighted mean difference
DVT: Deep venous thrombosis
